# Role for Non-Proteolytic Control of M-phase Promoting Factor Activity at M-phase Exit

**DOI:** 10.1371/journal.pone.0000247

**Published:** 2007-02-28

**Authors:** Vincenzo D'Angiolella, Luca Palazzo, Concetta Santarpia, Vincenzo Costanzo, Domenico Grieco

**Affiliations:** 1 Faculty of Biotechnological Sciences and Dipartimento di Biologia e Patologia Cellulare e Molecolare “L. Califano,” University of Napoli Federico II, Italy; 2 CEINGE Biotecnologie Avanzate, Napoli, Italy; 3 Department of Pathology, New York University, New York, United States of America; 4 Clare Hall Laboratories, London Research Institute, London, United Kingdom; Duke University, United States of America

## Abstract

M-phase Promoting Factor (MPF; the cyclin B-cdk 1 complex) is activated at M-phase onset by removal of inhibitory phosphorylation of cdk1 at thr-14 and tyr-15. At M-phase exit, MPF is destroyed by ubiquitin-dependent cyclin proteolysis. Thus, control of MPF activity via inhibitory phosphorylation is believed to be particularly crucial in regulating transition into, rather than out of, M-phase. Using the *in vitro* cell cycle system derived form Xenopus eggs, here we show, however, that inhibitory phosphorylation of cdk1 contributes to control MPF activity during M-phase exit. By sampling extracts at very short intervals during both meiotic and mitotic exit, we found that cyclin B1-associated cdk1 underwent transient inhibitory phosphorylation at tyr-15 and that cyclin B1-cdk1 activity fell more rapidly than the cyclin B1 content. Inhibitory phosphorylation of MPF correlated with phosphorylation changes of cdc25C, the MPF phosphatase, and physical interaction of cdk1 with wee1, the MPF kinase, during M-phase exit. MPF down-regulation required Ca^++^/calmodulin-dependent kinase II (CaMKII) and cAMP-dependent protein kinase (PKA) activities at meiosis and mitosis exit, respectively. Treatment of M-phase extracts with a mutant cyclin B1-cdk1AF complex, refractory to inhibition by phosphorylation, impaired binding of the Anaphase Promoting Complex/Cyclosome (APC/C) to its co-activator Cdc20 and altered M-phase exit. Thus, timely M-phase exit requires a tight coupling of proteolysis-dependent and proteolysis-independent mechanisms of MPF inactivation.

## Introduction

Rapid MPF activation is granted by an activation loop in which the cdc25C phosphatase removes inhibitory phosphorylations of cdk1 at thr-14 and tyr-15, while MPF stimulates cdc25C activity and lowers activity of wee1, the cdk1 tyr-15 kinase [1], [2]. At M-phase exit, MPF is destroyed by ubiquitin-dependent cyclin proteolysis inactivates (Peters 2002). Cyclin degradation is initiated by activation of the ubiquitin ligase APC/C associated with its co-activator Cdc20 (APC/C^Cdc20^) [3]. How timely activation of the degradation pathway is achieved is still incompletely understood. Several APC/C subunits and Cdc20 undergo MPF-dependent phosphorylation during M-phase [3]. Phosphorylation of APC/C stimulates its ubiquitin-ligase activity, however, phosphorylation of Cdc20 hampers binding to APC/C [3], [4], [5]. Thus, MPF activity may play both positive and negative actions on APC/C^Cdc20^ activation [6], [7]. Evidence suggests that interruption of the MPF activation loop may play a role for timing M-phase exit [8], [9]. To date, however, no direct evidence that the MPF activation loop is interrupted at M-phase exit has been provided. We set out to gain insight into this matter using the *in vitro* cell cycle system derived from Xenopus eggs.

## Results

### MPF activity loss at M-phase exit

**Figure 1:**
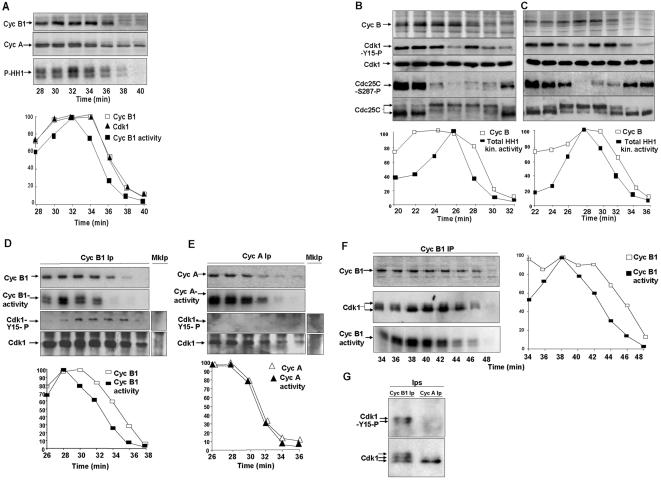
Cyclin B1, cyclin A abundance and MPF activity in cycling extracts. (*A*) Left panels, Cyclin B1 and cyclin A were visualised by immunoblot and total histone H1 kinase activity by autoradiograph of phosphorylated histone H1 (P-HH1) from samples of a cycling extract taken at the indicated time points during incubation at 23°C. Right panels, quantisation (percent of peak value) of cyclin B (open squares; from immunoblot in the left panel), cyclin B1-bound cdk1 (filled triangles; from cdk1 immunoblot of cyclin B1 immunoprecipitates; Ips) and histone H1 kinase (filled squares; autoradiographs of histone H1 kinase assayes from cyclin B1 Ips). (*B, C*) Left panels, cyclin B (extracts proteins autoradiograph; cyclin B position is indicated) and cdk1-phospho-tyr15 (Cdk1-Y15-P), cdk1 (Cdk1), cdc25C-phospho-ser-287 (Cdc25C-S287-P), cdc25C (Cdc25C) contents (immunoblot) from samples of two independent cycling extracts, incubated in the presence of [^35^S]methionine, taken at 2 min intervals. Right panels, quantisation of total extract histone H1 kinase (filled squares) and cyclin B (open squares) from the same samples. (*D*) Upper panels, cyclin B1, cyclin B1-associated kinase activity (Cyc B1-activity; phosphorylated histone H1 autoradiograph), cdk1-phospho-tyr-15 and cdk1 contents in cyclin B1 Ips from samples of an extract, incubated in the presence of [^35^S]methionine, taken across the mitosis-interphase transition. Lower graph, quantisation, expressed as percent of peak value, of cyclin B1 content (open squares) and cyclin B1-associated kinase activity (filled squares). (E) Upper panels, cyclin A (Cyc A) and cyclin A-associated kinase activity (Cyc A-activity; phosphorylated histone H1 autoradiograph), cdk1-phospho-tyr-15 and cdk1 contents in cyclin A Ips from the samples described in Fig 1D. Lower graph, quantisation, expressed as percent of peak value, of cyclin A content (open triangles) and cyclin A-associated kinase activity (filled triangles). (*F*) Samples from a cycling extracts, incubated in the presence of [^35^S]methionine, were taken at 2 min intervals. Samples were immunoprecipitated with an anti cyclin B1 antibody. Left panels, cyclin B1 (Cyc B1; autoradiograph), cyclin B1-associated cdk1 (Cdk1) was visualised by immunoblotting after resolving cyclin B1 Ips by long SDS-PAGE runs and cyclin B1-associated histone H1 kinase activity (Cyc B1 activity; autoradiograph). Right, quantisation of the autoradiographs of labelled cyclin B1 (open square) and phosphorylated histone H1 (filled square). From 34 to 38 min, the fastest migrating, dephosphorylated, cdk1 form accumulated as activity reached the maximum (38 min). Subsequently (40, 42, 44, 46 min), the slower migrating, phosphorylated, cdk1 forms reappeared as activity begun declining. (*G*) Cyclin B1 (Cyc B1 Ip) and cyclin A (Cyc A Ip) Ips from samples of a cycling extract taken 2 min after the cyclin B1-cdk1 activity peak. The cdk1-phospho-tyr-15 (Cdk1-Y15-P) and cdk1 were visualised by immunoblot after resolving the Ips by long SDS-PAGE runs. Since cyclin A is about 5 fold less abundant than cyclin B1 and cyclin A degradation was already started in the samples used, cyclin A Ips were from 6 times more extract sample than for cyclin B1 Ips to have comparable amounts of cdk1 on the blot.

**Figure 2:**
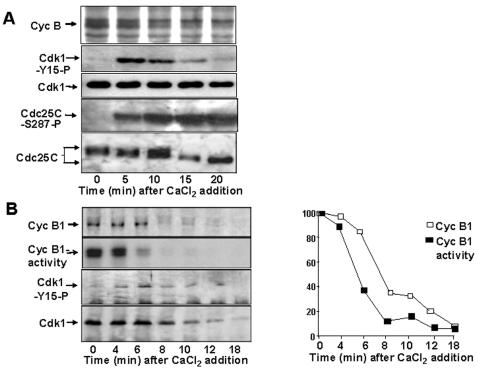
Cyclin B1 abundance and MPF activity in CSF-arrested extracts. (*A*) Cyclin B, cdk1-phospho-tyr15, cdk1, cdc25C-phospho-ser-287 and cdc25C contents from total extract samples of a CSF-arrested extract, pre-incubated with [^35^S]methionine, at the indicated time points after CaCl_2_ addition. (*B*) Left panels, cyclin B1, cyclin B1-associated kinase activity, cdk1-phospho-tyr15 and cdk1 contents in cyclin B1 Ips from CSF-arrested extract samples at the indicated time points after CaCl_2_ addition. Right, quantisation of cyclin B1 content (open squares), cyclin B1-associated kinase activity (filled squares) from cyclin B1 Ips.

Fluctuations in MPF activity and cyclin B concentration mark cell cycle progression during incubation of activated egg extracts at 23°C. At the end of M-phase, the fall in MPF activity relies on cyclin degradation [10]. By analying samples taken at short intervals (2 min) across the mitosis-to-interphase transition we repeatedly found that total egg extract's histone H1 kinase (a measure of MPF activity) [10], declined ahead of the cyclin B1 content and concomitantly with the decline of cyclin A (Fig. 1 A; upper panels; cyclins were detected from total extracts aliquots by immunoblot and total histone H1 kinase by autoradiograph of phosphorylated histone; P-HH1; note the decline in activity between 32 to 34 min and the stability of cyclin B1). By determining the amount of cyclin B1-associated cdk1 and kinase activity in cyclin B1 immunoprecipitates, we found a linear relationship in decline of cyclin B1-bound cdk1 and cyclin B1 content, thus excluding significant cyclin B1-cdk1 dissociation before degradation (Fig. 1A; graph; Cyc B1 and Cdk1). Nevertheless, cyclin B1-associated kinase activity declined ahead of the cyclin B1-cdk1 content (Fig. 1 A; graph; Cyc B1 activity). We asked whether inhibitory cdk1 tyr-15 phosphorylation could contribute to the observed loss of MPF activity. Samples of extracts, incubated in the presence of [^35^S]labelled methionine, were taken at 2 min intervals (Fig. 1 B, C). Probing total extracts aliquots with an anti cdk1-phospho-tyr-15 antibody showed that cdk1 underwent tyr-15 phosphorylation twice during the mitosis-to-interphase transition (Fig. 1 B, C; upper panels; shown are two independent extracts). After an initial decrease, that accompanied MPF activation and reached a minimum at the MPF activity peak (Fig. 1 B; 26 min; Fig. 1 C; 28 min), the cdk1-phopsho-tyr-15 signal transiently increased just around the time cyclin degradation begun (Fig. 1 B; 28 min; Fig. 1 C; 30, 32 min), to decrease again when substantial cyclin degradation was achieved (Fig. 1 B; 32 min; Fig. 1 C; 34 min). Determination of [^35^S]labelled cyclin and total histone H1 kinase activity also showed that activity fell more rapidly cyclin content (Fig. 1 B, C, graphs). We, next, determined the amount of [^35^S]labelled-cyclin, cdk1, cdk1-phospho-tyr-15 and kinase activity in cyclin B1 and cyclin A immunoprecipitates from extracts samples taken across the mitosis-to-interphase transition (Fig. 1 D, E). The decline in cyclin B1-associated kinase activity was accompanied by increased cyclin B1-associated cdk1-phospho-tyr-15 signal (Fig. 1 D). Cyclin A-associated kinase activity and cyclin A content decreased with parallel kinetics and begun when cyclin B1-associated kinase activity started to decline while cdk1-phospho-tyr15 was undetectable in cyclin A immunoprecipitates (Fig. 1 E). These findings were also revealed by changes in cdk1 migration after resolving cyclin B1 and cyclin A immunoprecipitates by longer SDS/PAGE runs (Fig. 1 F, G). At peak activity (Fig. 1 F; 38 min), cyclin B1-bound cdk1 was mostly in the fastest migrating form, dephosphorylated at inhibitory sites, while slower migrating cdk1 forms were present at earlier and later time points (Fig. 1. F; 40, 42, 44, 46). Cyclin A-bound cdk1, isolated from samples of an extract taken 2 min after MPF activity peak, did not reacted with an anti cdk1-phospho-tyr15 antibody nor showed slow migrating forms, unlike cyclin B1-bound cdk1 (Fig. 1 G). Similar changes in MPF activity, cdk1 phosphorylation and cyclin content were detected at meiotic metaphase II exit, analysing samples from non-activated eggs extracts after CaCl_2_ addition (to inactivate the calcium-sensitive Cytostatic Factor, CSF, that maintains metaphase II-arrest; Fig. 2 A) [11]. A wave of cdk1-tyr-15 phosphorylation was evident shortly after CaCl_2_ addition and analysis of cyclin B1 immunoprecipitates also revealed that activity fell ahead of cyclin content (Fig. 2 A, B).

### Sustained MPF activation loop delays M-phase exit

These data suggest that the MPF activation loop is interrupted at M-phase exit. Cdc25C inactivation correlates with its dephosphorylation at several sites and phosphorylation at the inhibitory site ser-287 [1], [12], [13]. At peak MPF activity, cdc25C-ser-287 and cdk1-tyr-15 phosphorylations were at minimum (Figs, 1 B, C; 26 min and 28 min respectively; Fig. 2 A; time 0). Cdc25C-ser-287 and cdk1-tyr-15 phosphorylations ensued when MPF activity begun declining (Figs, 1 B, C; Fig. 2 A). Cdc25C ser-287 phosphorylation increased as cell cycle progressed towards interphase, while cdk1-tyr-15 phosphorylation was lost along with the cyclin signal (Fig., 1 A, B; Fig. 2 A). Cdc25C also underwent a shift in migration on SDS/PAGE indicative of loss of multiple activating phosphorylations [1]. That only a small portion of cdc25C appeared to undergo inhibitory phosphorylation changes by the time of significant cdk1 phospho-tyr-15 reappearance may suggest that, in addition to dephosphorylation, cdk1-tyr-15 phosphorylation could possibly be regulated at M-phase exit (see below Fig. 5).

**Figure 3:**
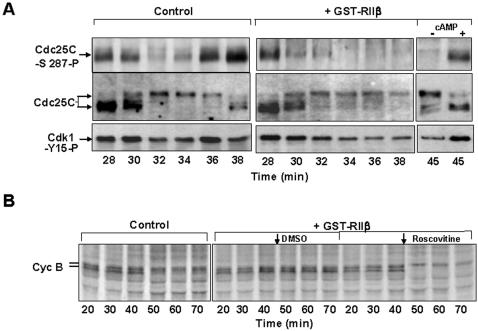
MPF hampers Cdc20-APC/C interaction. (*A*) Cdc25C-phospho-ser-287, cdc25C and cdk1-phospho-tyr15 contents during incubation time of a control cycling extract, treated with GST, and a portion of the same extract treated with GST-RIIβ (+GST-RIIβ) to inhibit PKA. To a portion of the GST-RIIβ-treated extract, cAMP (4 µM) was added at 40 min of incubation, to reactivate PKA, and samples taken at 45 min (-+cAMP). (*B*) Cyclin B stability during incubation time in a control extract and in portions of a GST-RIIβ-treated extract to which either roscovitine (2 µM; 1/30 extract volume), to inhibit cdk activity, or DMSO (1/30 extract volume), as control, were added.

**Figure 4:**
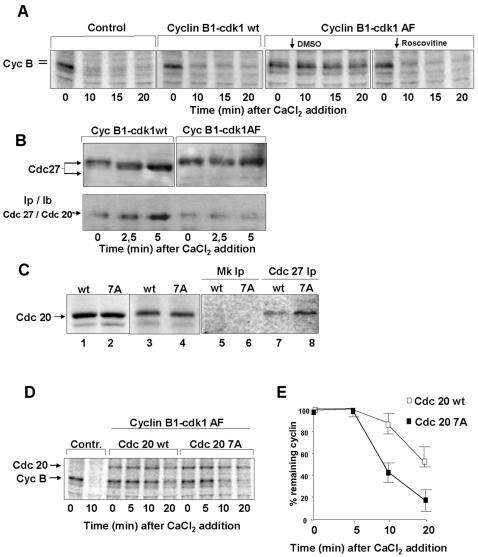
MPF hampers Cdc20-APC/C interaction through Cdc20 phopshorylation. (*A*) Cyclin B stability after CaCl_2_ addition in [^35^S]labelled CSF-arrested extract portions treated with buffer, as control, recombinant cyclin B1-cdk1wt or cyclin B1-cdk1AF complexes. To portions of the cyclin B1-cdk1AF-treated extract, either roscovitine (2 µM; 1/30 extract volume), or DMSO (1/30 extract volume) as control, were added 1 min after CaCl_2_. (*B*) Total Cdc27 and Cdc27-bound Cdc20 (IpCdc27/IbCdc20) from CSF-arrested samples treated with recombinant cyclin B1-cdk1wt and cyclin B1-cdk1AF complexes at the indicated time points after CaCl_2_ addition (the time 0 sample received no CaCl_2_). [^35^S]labelled Cdc20 wild type (wt) and a 7 phosphorylation sites mutant version (7A) were produced in reticulocyte lysates (lanes 1, 2). Labelled proteins were incubated with portions of a CHX-treated CSF-arrested extract for 30 min (lanes 3, 4). Cdc27 was, then, immunoprecipitated (Cdc 27 Ip) and the amount of bound wt (lane 7) and 7A (lane 8) Cdc20 detected by autoradiography. Lanes 5, 6, mock Ips (Mk Ip). (*C*) Portions of a [^35^S]labelled CSF-arrested extract were incubated for 40 min with mock (contr.), cdc20 wt or cdc20 7A programmed reticulocyte lysates in the presence of CHX. (*D*) The cdc20 wt- and cdc20 7A-treated portions where further incubated for 20 min with cyclin B1-cdk1AF. Then, aliquots were taken at the indicated time points after CaCl_2_ addition. Shown is an autoradiograph of [^35^S]labelled extracts proteins (the position of cyclin B is indicated). (*E*) Quantisation of remaining cyclin, expressed as percent, from cyclin B1-cdk1AF-treated extract portions in the presence of cdc20 wt (open squares) or cdc20 7A (filled squares). Error bars refer to variability within three independent experiments.

**Figure 5:**
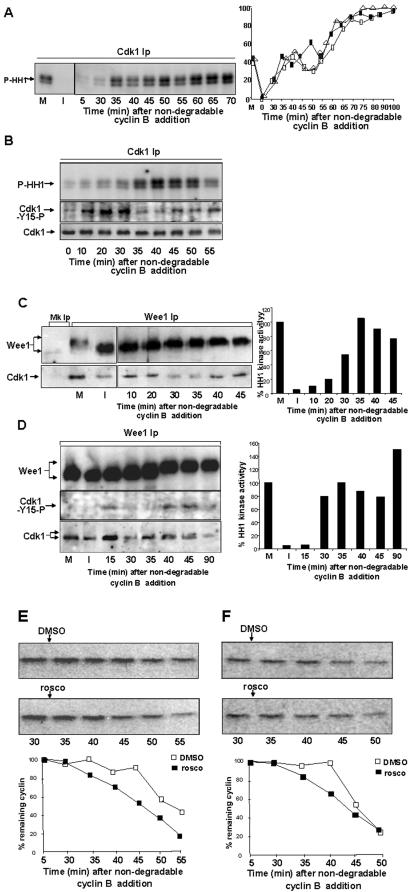
Wee1 and cdk1 physically interact in M-phase. (*A*) Histone H1 kinase activity in cdk1 Ips from samples of CSF-arrested extract (M), interphase extract (I; 40 min after CaCl_2_ and CHX additions) and from samples taken at the indicated time points after sea urchin ΔB (100nM) addition to an interphase extract. Left panel, phosphorylated histone H1 autoradiograph (P-HH1). Right panel, quantisation of cdk1 activity from three independent experiments. (*B*) Cdk1 activity, cdk1-phospho-tyr15 and cdk1 content in cdk1 Ips from extract samples taken at the indicated time points after ΔB addition. (*C, D*) Wee1 and cdk1 were immunoprecipitated from samples of CSF-arrested (M), interphase (I) extracts and interphase extract samples from the time of ΔB addition. Left panels, wee1 and wee1-associated cdk1 in wee1 Ips. Right, histone H1 kinase activity in cdk1 Ips. In (*D*) the lower part of the wee1 Ips immunoblot was first probed for cdk1-phospho-tyr15 and subsequently for cdk1. (*E, F*) Full-length, [^35^S]labelled, Xenopus cyclin B1 was added to two independent interphase extracts along with ΔB. The extracts were spilt into two portions and DMSO, as control, or roscovitine (10 µM) were added after 33 min incubation, samples were, then, taken at the indicated time points after ΔB addition. Shown are autoradiographs and quantisations of percent remaining [^35^S]labelled, full-length cyclin B1.

CaMKII and PKA appear relevant for direct cdc25C-ser-287 phosphorylation during unperturbed extract's cell cycle, CaMKII for the first mitotic interphase following CSF inactivation, while PKA for subsequent mitotic cycles [12], [13], [14], [15], [16]. PKA also stimulates a phosphatase activity, in egg extracts, that removes multiple activating cdc25C phosphorylations [14]. In cycling extract, addition of excess recombinant, glutathione-S-tranferase (GST)-fused, PKA regulatory subunit RIIβ (GST-RII), which inhibited PKA activity and delayed cyclin degradation as previously shown [15], also delayed the appearance of cdc25C-ser-287 and cdk1-tyr-15 phosphorylations, while cdc25C remained in its mitotic hyperphosphorylated state (Figs. 3 A, B). To determine whether sustained MPF activity had a role in delaying mitosis exit, cdk1 activity was lowered by adding the cdk inhibitor roscovitine to a PKA-inhibited extract. Cdk inhibition restored rapid cyclin degradation (Fig. 3 B). Thus, when the MPF activation loop is sustained by PKA inhibition, cyclin degradation is delayed in an MPF-dependent fashion. In CSF-arrested extracts, cdc25C-ser-287 and cdk1-tyr-15 phosphorylations were induced rapidly after calcium addition and prevented by treatment with the CaMKII inhibitor peptide CaMKII Ntide (Fig. S1).

### Cdk1AF delays M-phase exit and hinders Cdc20-APC/C interaction

It has been recently published that cycling extracts forced to use a mutant cdk1AF (cdk1AF; thr 14>ala, and tyr 15>phe), refractory to inhibitory phosphorylation, enter mitosis with slower kinetics than control extracts and are delayed in exiting mitosis [17]. However, in that report the defects in M-phase exit were ascribed to the slower onset of M-phase caused by cdk1AF rather than to a direct interference of cdk1AF with the M-phase exit mechanisms. To determine whether cdk1AF had a direct effect on M-phase exit, regardless of how the mitotic state was reached, we treated a CSF-arrested extract, that is already naturally arrested in M-phase, with a cyclin B1-cdk1AF complex and monitored cyclin degradation kinetics upon calcium addition. We found that cyclin degradation was delayed in cyclin B1-cdk1AF-treated but not in cyclin B1-cdk1wt-treated extract, as control (Fig. 4 A). Rapid cyclin degradation was, however, restored in the cyclin B1-cdk1AF-treated extract by lowering cdk activity with roscovitine (Fig. 4 A). Although only slightly delayed degradation of cyclin A, cyclin B1-cdk1AF treatment significantly affected the morphological changes of added nuclei hindering the metaphase-to-anaphase transition as well as subsequent chromosome disjunction (Figs. S2, S3).

We asked why interruption of the MPF activation loop was required for timely activation of cyclin degradation. In this system, cyclin degradation tightly relies on the ubiquitin ligase Anaphase Promoting Complex/Cyclosome (APC/C) in association with its co-activator Cdc20 and assembly of APC/C^Cdc20^ is required for efficient ubiquitin ligase activity [3], [18]. By analyzing the amount of Cdc20 that co-immunoprecipitated with Cdc27, an APC/C component, we found that CSF-arrested extracts already contained some APC/C-Cdc20 complex, but APC/C-Cdc20 interaction was transiently stimulated after calcium addition (Fig. S4 and Fig. 4 B; cyc B1-cdk1wt). Treatment with cyclin B1-cdk1AF delayed induction of APC/C-cdc20 interaction after calcium addition (Fig. 4 B). APC/C and Cdc20 undergo phosphorylation in M-phase. While APC/C phosphorylation stimulates its ubiquitin-ligase activity and affinity for cdc20 [3], cdk-dependent phosphorylation of Cdc20 appears to hamper binding to APC/C, thus restraining APC/C^Cdc20^ activation [4], [5]. To determine whether Cdc20 phosphorylation affected binding to APC/C in egg extracts, CSF extracts were incubated with *in vitro* translated wild type human Cdc20 and a mutant Cdc20 version, non-phosphorylatable at 7 cdk1 consensus sites (Cdc207A) [4], for 40 min, to allow Cdc20 phosphorylation (that caused retarded mobility on SDS/PAGE; Fig. 4 C) [5]. After incubation, more Cdc207A than wild type Cdc20 could be recovered in Cdc27 immunoprecipitates (Fig. 4 C), indicating that Cdc20 phosphorylation hampered APC/C-Cdc20 interaction in the extracts. Indeed, treatment of extracts with Cdc207A, rather than wild type Cdc20, partly reversed the cyclin B1-cdk1AF-induced delay in cyclin degradation (Fig. 4 D, E). Thus, loss of MPF-dependent Cdc20 phosphorylation helps APC/C^Cdc20^ activation [4], [5].

### Wee1 and cdk1 physically interact in M-phase

By our analysis it cannot be excluded that the pathways leading to MPF inactivation via inhibitory phosphorylation take the upper hand only after initial degradation of small cyclin B amounts. In addition, the fact that cyclin degradation is activated when cdk1 activity is sustained by non-degradable cyclin B [19] suggests that a reduction in MPF activity is not absolutely required to initiate cyclin degradation. To gain further insight into this aspect, we assayed cdk1 activity in cdk1 immunoprecipitates from samples taken at 5 min intervals from the time of recombinant non-degradable sea urchin cyclin B (ΔB; at 100 nM) addition to interphase extracts. We found that, after the already described lag of 10–20 min [20], cdk1 activity did not increase continuously during incubation (Fig. 5 A). Shortly after reaching M-phase activity levels (Fig. 5 A; around 35–40 min after ΔB addition), activity underwent fluctuations with decrements up to 20–25% relatively to M-phase activity (Fig. 5 A; 40 to 60 min). Later, it further increased with relatively steady increments (Fig. 5 A; from 60 min on). As previously demonstrated [20], cdk1-tyr-15 phosphorylation was stimulated shortly after ΔB addition and decreased as cdk1 activity reached M-phase activity levels (Fig. 5 B, 10 to 40 min). Subsequently, the fluctuation in cdk1 activity was accompanied by a relative increase in cdk1-tyr-15 phosphorylation (albeit rather lower than that observed at early time points; Fig. 5 B, 45–50–55 min). Cdc25C has been reported to be hyperphosphorylated at activating sites and non-phosphorylated at ser-287 [21] under ΔB-induced M-phase arrest, making unlikely its involvement in cdk1 phosphorylation control under these conditions. Wee1 activity is believed to be down-regulated in M-phase by cdk1-dependent phosphorylations [22]. Recent evidence indicated that wee1 undergoes phosphorylation at ser-549 in M-phase of unperturbed cell cycle as well as under ΔB conditions [21]. However, by *in vitro* kinase assays, wee1-ser-549 phosphorylation did not increase wee1 activity and wee1 isolated from interphase was slightly more active than that isolated from M-phase [21]. What mechanism could, then, lead to mitotic cdk1-tyr-15 phosphorylation under ΔB conditions? We investigated whether wee1 and MPF could physically interact in M-phase, as recently shown for the budding yeast wee1 homolog swe1 [23], providing a possible mechanism for mitotic cdk1-tyr-15 phosphorylation. Indeed, cdk1 was detected in wee1 immunoprecipitates from CSF-arrested extracts, while little cdk1 co-precipitated with wee1 from interphase samples (Fig 5 C). Under ΔB conditions, wee1-cdk1 iteraction was detectable 10–20 min after ΔB addition, when cdk1 activity was still low (Fig. 5 C). Subsequently, the complex significantly dissociated (Fig. 5 C; 20–35 min). However, after cdk1 regained M-phase activity levels and then started to fluctuate, wee1-cdk1 interaction was regained (Fig. 5 C; 35–45 min). Probing wee1 immunoprecipitates for cdk1 phospho-tyr-15 showed that the cdk1-phospho-tyr-15 signal was detectable at early (interphase; Fig. 5 D; 10 min) and, more pronouncedly, at later (M-phase; Fig. 5 D; 40–45 min) time points after ΔB addition, further indicating that wee1-cdk1 interaction is accompanied by cdk1-tyr-15 phosphorylation. When cdk1 activity further increased, upon prolonged incubation, cdk1 appeared to dissociate from wee1 (Fig. 5 D; 90 min), possibly because of wee1 hyperphosphorylation [23]. Preliminary results indicate that wee1-cdk1 interaction is also detected during unperturbed M-phase exit in cycling extracts (not shown).

Under our experimental conditions, degradation of full-length cyclin initiated 40–45 min from addition of non-degradable cyclin B (Fig. 5 E). To determine whether ΔB-cdk1 activity affected cyclin degradation timing, we inhibited cdk1 activity after significant cdk1 activity had been reached following ΔB addition (33 min; Figs. 5 E, F) and monitored full-length cyclin B degradation. Cdk1 inhibition slightly but consistently accelerated initiation of degradation (Figs. 5 E, F). Toghether these data indicate that the cdk1-tyr-15 phosphorylation state at M-phase exit may also be regulated via wee1-cdk1 interaction and indicate that cyclin B-cdk1 activity can affect the cyclin degradation pathway even under conditions in which degradation is prevented.

Further investigation will be required to establish the role for phosphorylations [21], [22], [23] in the wee1-cdk1 interaction and in wee1 activity towards wee1-bound cdk1 as well as the domains involved in binding and other potential M-phase aspects for which interaction is relevant [24].

## Discussion

Our data show that timely completion of M-phase requires proteolysis-independent control of MPF activity. Nevertheless, M-phase exit strictly relies on proteolysis and phosphorylation-dependent MPF down-regulation is insufficient to lower MPF activity to the levels required to exit M-phase if proteolysis cannot take over. We propose that proteolysis and inhibitory cdk1 phosphorylation are interconnected in a MPF inactivation loop for M-phase exit. Initial partial, phosphorylation-dependent, loss of MPF activity stimulates APC/C^Cdc20^ activation. MPF degradation further weakens the MPF activation loop promoting MPF inhibitory phosphorylation. Consequent additional MPF activity loss further helps APC/C^Cdc20^ activation. We cannot exclude that degradation of a small pool of cyclin initiates the loop. However, the observation that MPF binds to wee1 in M-phase even in the absence of cyclin degradation (Fig. 5) further witnesses that a tight coupling of proteolysis-independent and proteolysis-dependent mechanisms of MPF inactivation coordinate M-phase exit.

## Materials and Methods

### Egg extracts and immunoprecipitations

Xenopus egg extracts were prepared essentially as described [10]. Where indicated extracts were incubated in the presence of [^35^S]methionine (400 mCi/ml; Amersham). Histone H1 kinase assays were performed as described [15] and quantified as percentage from densitometry values of phosphorylated histone H1 autoradiographs. Roscovitine and CaMKII Ntide peptide were purchased from Calbiochem. Immunoprecipitations (Ips) were performed as follows: 5 to 40 ml of extracts samples were diluted in 250 or 500 ml ice cold buffer containing 15 mM Hepes pH 7.4, 150 mM NaCl, 0.1% NP40 and phsosphatase and protease inhibitors (Sigma). Samples, pre-cleared with protein A-+G-sepharose, were incubated with 2 mg antibody for 1 hour on ice, mixed with 20 ml of 50% protein A+G-sepharose suspension and incubated with rotation at 4°C for 1 hour. Bead pellets were washed once with low (150 mM NaCl) and high (400 mM NaCl) salt buffer and once again with low (150 mM NaCl). At the final wash, when needed, Ips were divided into various portions for to analyse activity or protein content. Mock precipitations were performed with non-immune Ig from time 0 samples. Cdc20-Cdc27 co-Ips were performed according to previously published methods [25]; briefly: at the end of each treatment, 50 ml extract samples were quickly frozen in liquid nitrogen. Samples were subsequently thawed in 20 vol. of XB/EB (1/1) [10] and 100 nM okadaic acid (Roche). Samples were incubated with 1 µg of anti Cdc27 antibody for 1 hours on ice, mixed with 20 ml of 50% protein A+G-sepharose suspension and further incubated with rotation at 4°C for 1 hour. Bead pellets were washed 4 times with XB/EB (1/1) and processed for immunoblot analysis according to standard procedures. Co-Ips between cdc27 and exogenous, labelled, cdc20 wt and 7A were performed similarly. Nuclear morphology was analysed as described [26]. Briefly, demembranated sperm nuclei (300/µl extract) were allowed to undergo DNA replication by 120 min incubation with interphase extracts. Nuclei were then moved into metaphase by incubating one part of the interphase extract (+nuclei) with two parts of a CSF-arrested extract. Incubations with recombinant proteins were performed as indicated in legends. Anti Xenopus cyclin B1 and cyclin A, antibodies were a generous gift of J. Maller; anti Xenopus Cdc20/Fizzy and anti cdc25C-phospho-ser 287 were a generous gift of M. Dasso, A. Arnoutov and J. Ruderman; anti Xenopus cdc25C was a gift of J. Gannon. Other antibodies were purchased from Santa Cruz Biotechnology, Zymed and Transduction Laboratories.

### Recombinant proteins

Cdc20 wt and 7A plasmids were a generous gift of A. Hershko. Proteins were *in vitro* translated in the presence of [^35^S]labelled methionine and cysteine (promix; Amersham) using a T7-TNT coupled system (Promega). Production and purification of GST-human cyclin B1-cdk1wt and GST-human cyclin B1-cdk1AF kinases frombaculovirus-infected Sf9 cells was performed as described [26]. Egg extracts were pre-incubated with either buffer, recombinant cyclin B1-cdk1wt or cyclin B1-cdk1AF for 20 min at 30°C before CaCl_2_ addition. Equal amounts to the endogenous level of cyclin B1-cdk1 activity were added of recombinant enzymes.

## Supporting Information

Figure S1 Cdc25C and cdk1 phosphorylation changes are induced upon CaCl2 addition to CSF-arrested extracts and prevented by CaMKII inhibition. Cdc25C and cdc25C-phospho-ser-287 (Cdc25; two exposures of the immunoblot are shown; the asterisks mark non-specific signals) and cdk1-phospho-tyr15 contents from portions of a CSF-arrested extract, pre-incubated for 20 min with buffer, as control, or with the CaMKII inhbitor peptide CaMKII Ntide (0.5 mM), taken at the indicated time points after CaCl2 addition.
(0.16 MB TIF)

Figure S2 Cyclin B and cyclin A stability in cyclin B1-cdk1AF-treated CSF-arrested extracts. Portions of a CSF-arrested extract were pre-incubated for 20 min at 23 degrees C with buffer or recombinant cyclin B1-cdk1AF. Then, CaCl2 was added and samples taken at the indicated time points. Upper panels, Cyclin B1 and cyclin A were detected by immunoblot. Lower left panel, densitometric quantisation (from immunoblot signals; expressed as percent of peak value) of the cyclin A content in control (filled squares; Cyc A cont.-ertx.) and cyclin B1-AF-treated (open squares; Cyc A AF-ertx.) extrac portions. Lower right panel, densitometric quantisation (from immunoblot signals; expressed as percent of peak value) of the cyclin B1 content in control (filled squares; Cyc B1 cont.-ertx.) and cyclin B1-AF-treated (open squares; Cyc B1 AF-ertx.) extract portions. Error bars refer to variability within three independent experiments.
(0.10 MB TIF)

Figure S3 Nuclear morphology in cyclin B1-cdk1AF-treated CSF-arrested extracts. Demembranated sperm nuclei (300/µl extract) were allowed to undergo DNA replication by 120 min incubation with interphase extracts. Nuclei were then moved into metaphase by incubating one part of the interphase extract (+nuclei) with two parts of a CSF-arrested extract for 50 min. After incubation, portions were treated either with buffer (control) or cyclin B1-AF and further incubated at 23 degrees C for 20 min. Samples were fixed at the indicated time points after CaCl2 addition and visualised by Hoechst staining. A portion of the cyclin B1-cdk1AF-treated extract also received roscovitine (2 µM in DMSO) 1 min after CaCl2 addition (Cyclin B1-AF+Rosco). DMSO addition alone did not affect morphological changes in cyclin B1-AF-treated extracts (not shown).
(0.08 MB TIF)

Figure S4 Cdc20-Cdc27 interaction in CSF-arrested extracts. Cdc27 was immunoprecipitated (Cdc27 Ip) and bound Cdc20 visualised by immunoblot to from untreated CSF-arrested samples, pre-incubated for 20 min at 23 degrees C before CaCl2 addition to maintain similar conditions to the experiments with recombinant cyclin B1-cdk1 complexes. Samples were taken at the indicated time points after CaCl2 addition. Mock precipitations (MkIp) were performed with non-immune Ig from time 0 samples
(0.05 MB TIF)

## References

[1] Hoffmann I, Clarke PR, Marcote MJ, Karsenti E, Draetta G (1993). Phosphorylation and activation of human cdc25-C by cdc2-cyclin B and its involvement in the self-amplification of MPF at mitosis.. EMBO J.

[2] Dunphy WG (1994). The decision to enter mitosis.. Trends Cell Biol.

[3] Peters JM (2002). The anaphase-promoting complex: proteolysis in mitosis and beyond.. Mol Cell.

[4] Yudkovsky Y, Shteinberg M, Listovsky T, Brandeis M, Hershko A (2000). Phosphorylation of Cdc20/fizzy negatively regulates the mammalian cyclosome/APC in the mitotic checkpoint.. Biochem Biophys Res Commun.

[5] D'Angiolella V, Mari C, Nocera D, Rametti L, Grieco D (2003). The spindle checkpoint requires cyclin-dependent kinase activity.. Genes & Dev.

[6] D'Angiolella V, Grieco D (2004). Attach first, then detach. A role for cyclin B- dependent kinase in coordinating proteolysis wirh spindle assembly.. Cell Cycle.

[7] Ciliberto A, Lukacs A, Toth A, Tyson JJ, Novak B (2005). Rewiring the Exit from Mitosis.. Cell Cycle.

[8] D'Angiolella V, Costanzo V, Gottesman ME, Avvedimento EV, Gautier J (2001). Role for cyclin-dependent kinase 2 in mitosis exit.. Curr Biol.

[9] Wolfe BA, Gould K L (2004). Inactivating Cdc25, mitotic style.. Cell Cycle.

[10] Murray AW, Kirschner MW (1989). Cyclin synthesis drives the early embryonic cell cycle.. Nature.

[11] Tunquist BJ, Maller JL (2003). Under arrest: cytostatic factor (CSF)- mediated metaphase arrest in vertebrate eggs.. Genes & Dev.

[12] Duckworth BC, Weaver JS, Ruderman JV (2002). G2 arrest in Xenopus oocytes depends on phosphorylation of cdc25 by protein kinase A.. Proc Natl Acad Sci U S A.

[13] Hutchins JR, Dikovskaya D, Clarke PR (2003). Regulation of Cdc2/cyclin B activation in Xenopus egg extracts via inhibitory phosphorylation of Cdc25C phosphatase by Ca(2+)/calmodulin-dependent protein kinase II.. Mol Biol Cell.

[14] Grieco D, Avvedimento VE, Gottesman ME (1994). A role for cAMP- dependent protein kinase in early embryonic divisions.. Proc. Natl. Acad. Sci..

[15] Grieco D, Porcellini A, Avvedimento EV, Gottesman ME (1996). Requirement for cAMP-PKA pathway activation by M phase-promoting factor in the transition from mitosis to interphase.. Science.

[16] Lorca T, Cruzalegui FH, Fesquet D, Cavadore JC, Mery J (1993). Calmodulin-dependent protein kinase II mediates inactivation of MPF and CSF upon fertilization of Xenopus eggs.. Nature.

[17] Pomerening JR, Kim SY, Ferrell JE (2005). Systems-level dissection of the cell-cycle oscillator: bypassing positive feedback produces damped oscillations.. Cell.

[18] Lorca T, Castro A, Martinez AM, Vigneron S, Morin N (1998). Fizzy is required for activation of the APC/cyclosome in Xenopus egg extracts.. EMBO J.

[19] Luca FC, Shibuya EK, Dohrmann CE, Ruderman JV (1991). Both cyclin A delta 60 and B delta 97 are stable and arrest cells in M-phase, but only cyclin B delta 97 turns on cyclin destruction.. EMBO J.

[20] Solomon MJ, Glotzer M, Lee TH, Philippe M, Kirschner MW (1990). Cyclin activation of p34cdc2.. Cell.

[21] Stanford JS, Ruderman JV (2005). Changes in Regulatory Phosphorylation of Cdc25C Ser287 and Wee1 Ser549 during Normal Cell Cycle Progression and Checkpoint Arrests.. Mol Biol Cell.

[22] Kim SY, Song EJ, Lee KJ, Ferrell JE (2005). Multisite M-phase phosphorylation of Xenopus Wee1A.. Mol Cell Biol.

[23] Harvey SL, Charlet A, Haas W, Gygi S P, Kellogg DR (2005). Cdk1- dependent regulation of the mitotic inhibitor Wee1.. Cell.

[24] Stumpff J, Kellogg DR, Krohne KA, Su TT (2005). Drosophila Wee1 interacts with members of the gammaTURC and is required for proper mitotic- spindle morphogenesis and positioning.. Curr Biol.

[25] Arnaoutov A, Dasso M (2003). The Ran GTPase regulates kinetochore function.. Dev Cell.

[26] Costanzo V, Robertson K, Ying CY, Kim E, Avvedimento E (2000). Reconstitution of an ATM-dependent checkpoint that inhibits chromosomal DNA replication following DNA damage.. Mol Cell.

