# Supplementary figures and images for: Role for Non-Proteolytic Control of M-phase Promoting Factor Activity at M-phase Exit
Source: PLoS One. 2007 Feb 28;2(2):e247. doi: 10.1371/journal.pone.0000247 (PMC1803016; doi:10.1371/journal.pone.0000247)

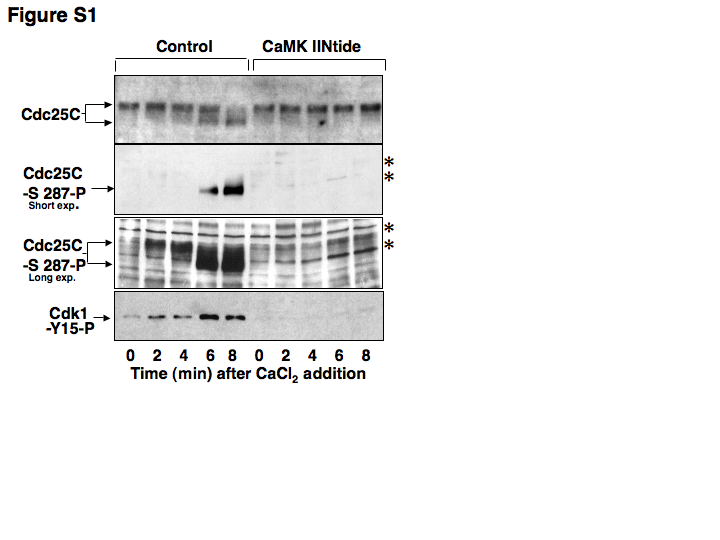

Supplement: Figure S1 — Cdc25C and cdk1 phosphorylation changes are induced upon CaCl2 addition to CSF-arrested extracts and prevented by CaMKII inhibition. Cdc25C and cdc25C-phospho-ser-287 (Cdc25; two exposures of the immunoblot are shown; the asterisks mark non-specific signals) and cdk1-phospho-tyr15 contents from portions of a CSF-arrested extract, pre-incubated for 20 min with buffer, as control, or with the CaMKII inhbitor peptide CaMKII Ntide (0.5 mM), taken at the indicated time points after CaCl2 addition. (0.16 MB TIF) [file pone.0000247.s001.tif]

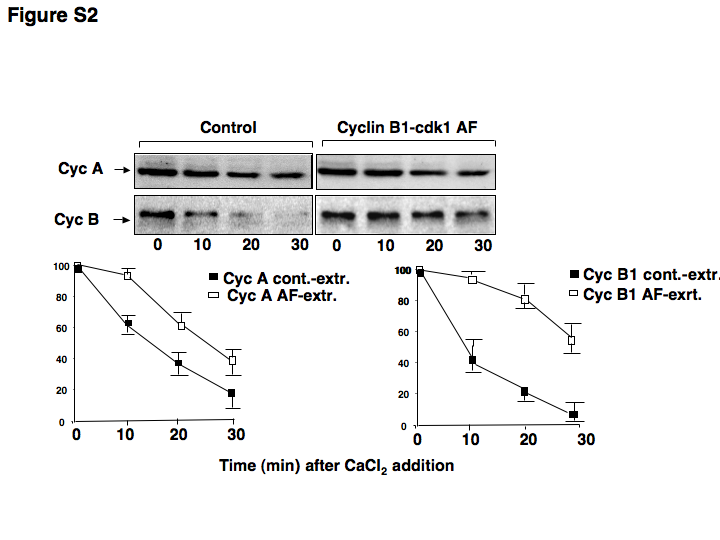

Supplement: Figure S2 — Cyclin B and cyclin A stability in cyclin B1-cdk1AF-treated CSF-arrested extracts. Portions of a CSF-arrested extract were pre-incubated for 20 min at 23 degrees C with buffer or recombinant cyclin B1-cdk1AF. Then, CaCl2 was added and samples taken at the indicated time points. Upper panels, Cyclin B1 and cyclin A were detected by immunoblot. Lower left panel, densitometric quantisation (from immunoblot signals; expressed as percent of peak value) of the cyclin A content in control (filled squares; Cyc A cont.-ertx.) and cyclin B1-AF-treated (open squares; Cyc A AF-ertx.) extrac portions. Lower right panel, densitometric quantisation (from immunoblot signals; expressed as percent of peak value) of the cyclin B1 content in control (filled squares; Cyc B1 cont.-ertx.) and cyclin B1-AF-treated (open squares; Cyc B1 AF-ertx.) extract portions. Error bars refer to variability within three independent experiments. (0.10 MB TIF) [file pone.0000247.s002.tif]

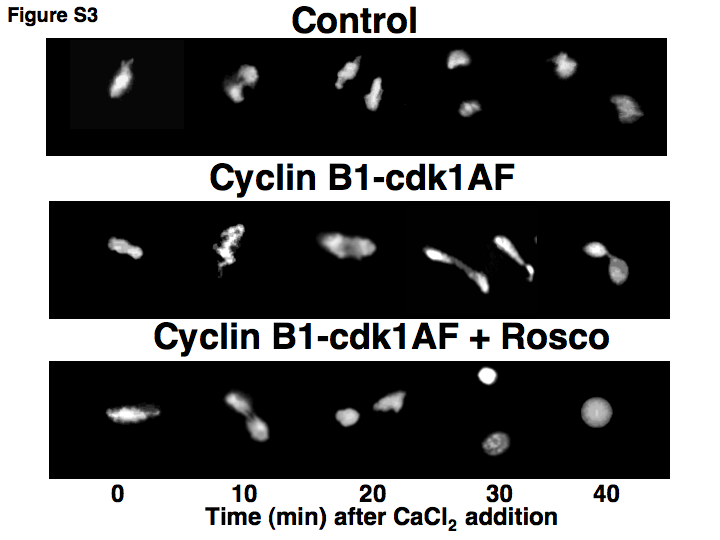

Supplement: Figure S3 — Nuclear morphology in cyclin B1-cdk1AF-treated CSF-arrested extracts. Demembranated sperm nuclei (300/µl extract) were allowed to undergo DNA replication by 120 min incubation with interphase extracts. Nuclei were then moved into metaphase by incubating one part of the interphase extract (+nuclei) with two parts of a CSF-arrested extract for 50 min. After incubation, portions were treated either with buffer (control) or cyclin B1-AF and further incubated at 23 degrees C for 20 min. Samples were fixed at the indicated time points after CaCl2 addition and visualised by Hoechst staining. A portion of the cyclin B1-cdk1AF-treated extract also received roscovitine (2 µM in DMSO) 1 min after CaCl2 addition (Cyclin B1-AF+Rosco). DMSO addition alone did not affect morphological changes in cyclin B1-AF-treated extracts (not shown). (0.08 MB TIF) [file pone.0000247.s003.tif]

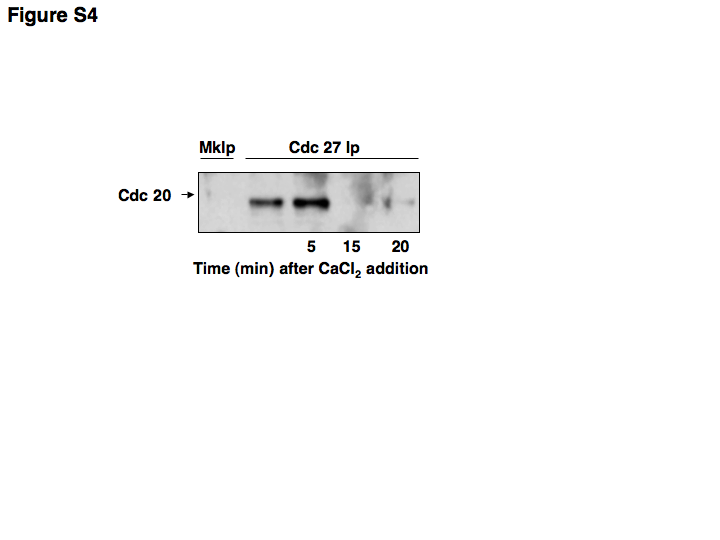

Supplement: Figure S4 — Cdc20-Cdc27 interaction in CSF-arrested extracts. Cdc27 was immunoprecipitated (Cdc27 Ip) and bound Cdc20 visualised by immunoblot to from untreated CSF-arrested samples, pre-incubated for 20 min at 23 degrees C before CaCl2 addition to maintain similar conditions to the experiments with recombinant cyclin B1-cdk1 complexes. Samples were taken at the indicated time points after CaCl2 addition. Mock precipitations (MkIp) were performed with non-immune Ig from time 0 samples (0.05 MB TIF) [file pone.0000247.s004.tif]
